# Melanoma Metastasizing to the Small Intestine: A Case Report Illustrating Symptomatic and Asymptomatic Involvement

**DOI:** 10.7759/cureus.608

**Published:** 2016-05-13

**Authors:** FNU Asad-Ur-Rahman, Aamer Abbass, Umair Majeed, Udayakumar Navaneethan

**Affiliations:** 1 Internal Medicine Residency, Florida Hospital-Orlando; 2 Center for Interventional Endoscopy, Florida Hospital-Orlando

**Keywords:** melanoma, acute abdomen, perforation, duodenum, jejunum

## Abstract

Symptomatic gastrointestinal (GI) involvement of melanoma is rare, however, it is a frequent autopsy finding in patients with primary cutaneous melanoma. We present a case of metastatic cutaneous melanoma with initial asymptomatic jejunal involvement as found on a positron emission tomography (PET) scan, with subsequent duodenal perforation.

A 69-year-old man presented to the hospital with a three-week history of worsening headache, dizziness, and vomiting with a history of Clark level III malignant melanoma that was completely excised from the right flank three years ago at the hospital. A magnetic resonance image of his brain revealed a subacute right-sided cerebellar hemorrhage adjacent to a 1-cm nodule. He underwent a right suboccipital craniomy with resection; the biopsy of which revealed metastatic malignant melanoma. A staging positron emission tomography (PET) scan revealed areas of increased uptake of fludeoxyglucose (FDG) in the left lower lung and left upper quadrant of the abdomen abutting the small bowel. Subsequent enteroscopy revealed a 40-mm cratered jejunal ulcer with heaped edges; the biopsy of which also revealed malignant melanoma. Since he had widespread disease, abdominal surgery was deferred, and treatment with ipilimumab and radiotherapy to the brain was initiated. He presented three months later with acute abdominal pain and diarrhea. A computed tomography scan of his abdomen revealed free peritoneal air, and an exploratory laparotomy revealed a mass at the antimesenteric border of the duodenum with a biopsy consistent with melanoma. The perforated area was resected and an end-to-end anastomosis was performed. Unfortunately, our patient had a postoperative intracranial hemorrhage and was referred to palliative care.

Our case portrays how malignant melanoma may metastasize insidiously and widely and present as a catastrophe. Melanoma involvement in the GI tract is a poor prognostic marker. Our case offers a unique illustration of both the occult and manifest gastrointestinal involvement of melanoma and underscores the importance of clinical suspicion in patients with a history of melanoma who present with unexplained GI symptoms.

## Introduction

Symptomatic gastrointestinal (GI) involvement of melanoma is rare (one to four percent) but is a frequent autopsy finding (60%) in patients who have a primary cutaneous melanoma [1]. This demonstrates its insidious nature of progression that merits a high index of suspicion in patients with abdominal complaints with a diagnosis of malignant melanoma. The small bowel is the most frequent site of metastases in the GI tract, which may be attributed to its rich vascularity [2]. We present a case of metastatic cutaneous melanoma with initial asymptomatic involvement of the jejunum as found on a positron emission tomography (PET) scan and enteroscopy. The patient subsequently developed duodenal perforation due to further metastatic spread. Informed consent was obtained from the patient for this study.

## Case presentation

A 69-year-old man presented to the hospital with a three-week history of worsening headache, dizziness, and vomiting with a history of Clark level III malignant melanoma that was completely excised from the right flank three years prior to his arrival at the hospital. He had no associated syncope or seizures. He denied any abdominal pain, diarrhea, melena, or hematemesis.

Physical examination revealed right-sided cerebellar ataxia and nystagmus with a lateral gaze on the right, but no demonstrable cranial nerve abnormality or focal body weakness or numbness. He also did not have any abdominal tenderness or visceromegaly.

**Figure 1:**
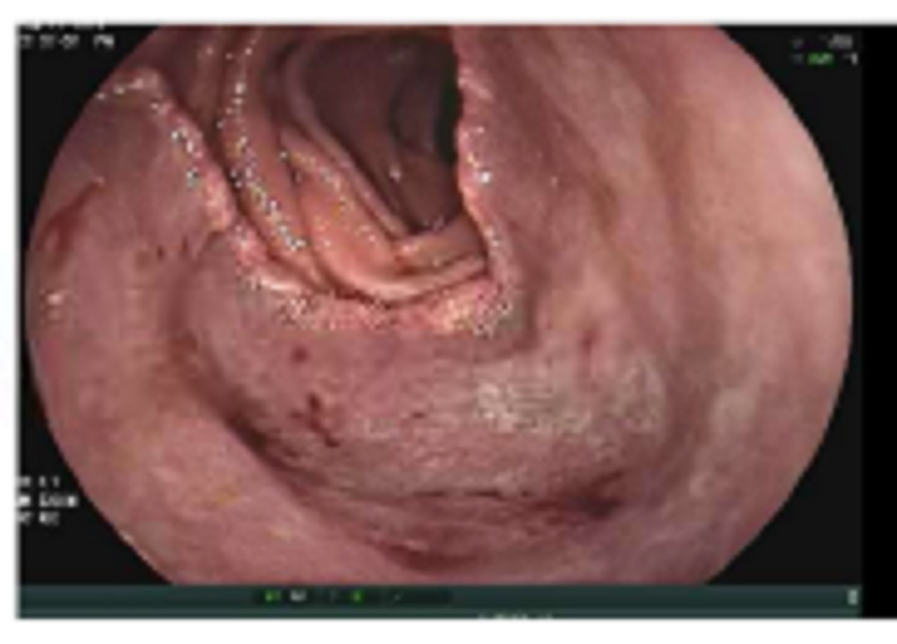
Enteroscopy revealing a non-bleeding, large cratered ulcer measuring 40 mm in largest dimension with heaped edges in the proximal jejunum, occupying >75% of the lumen.

Laboratory studies revealed normal complete blood count and basic metabolic panel. A magnetic resonance image (MRI) of his brain revealed a 4 X 3.3 X 3 cm, subacute right-sided cerebellar hemorrhage adjacent to a 1-cm heterogeneous nodule. There was another 1-cm suspicious lesion on the right parietal hemisphere with minimal enhancement. He underwent a right suboccipital craniotomy with resection. A biopsy of the lesion was found positive for malignant melanoma with strongly positive S100, Melan-A, and HMB45 stains. As part of the staging work-up, a PET scan was done and that revealed an increased FDG uptake in the left lower lung and the upper left quadrant of the abdomen abutting the small bowel. The patient underwent enteroscopy to assess the underlying lesion, revealing a large, non-bleeding, cratered ulcer measuring 40 mm in its largest diameter with heaped edges in the proximal jejunum and occupying >75% of the lumen (Figure 1).

**Figure 2:**
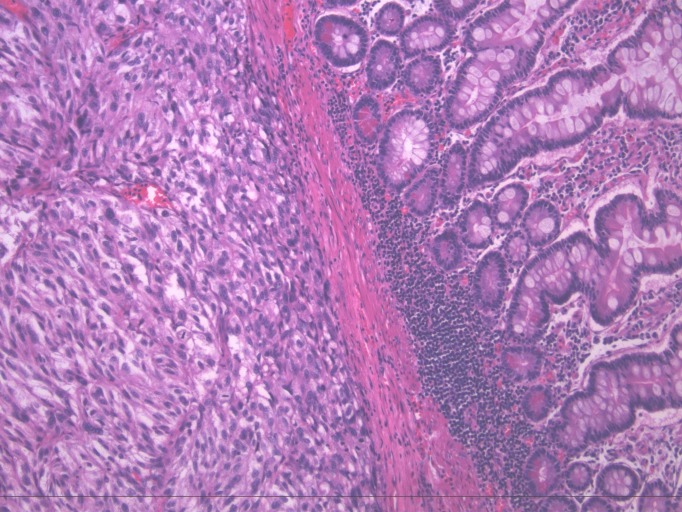
Biopsy of the ulcer with hematoxylin and eosin stain, depicting infiltration in a nesting fashion on the left side consistent with metastatic melanoma. Normal mucosa evident on the right.

**Figure 3:**
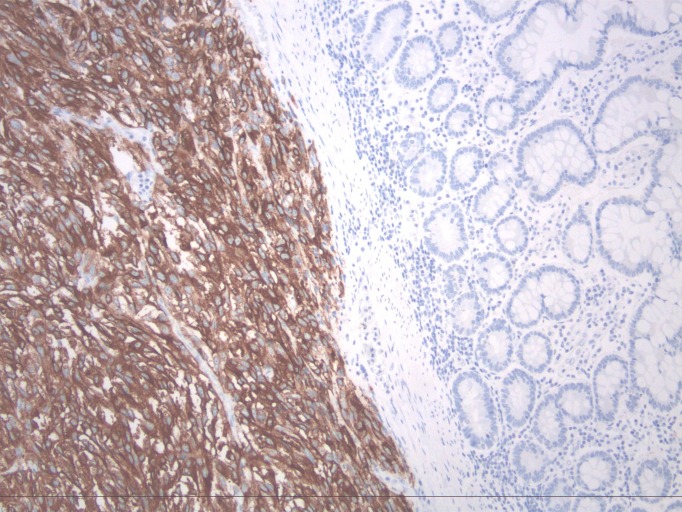
Similar section to Figure 2. HMB-45 (melanoma associated antigen) immunohistochemistry positive (brown stain) in melanoma cells on the left compared to normal cells on the right.

A biopsy of the ulcer revealed metastatic melanoma cells (Figure 2) that stained strongly positive for Melan A (Figure 3).

A computed tomography (CT) guided lung biopsy of the left lower lobe mass measuring 9.9 X 8.0 cm, also revealed metastatic melanoma.

**Figure 4:**
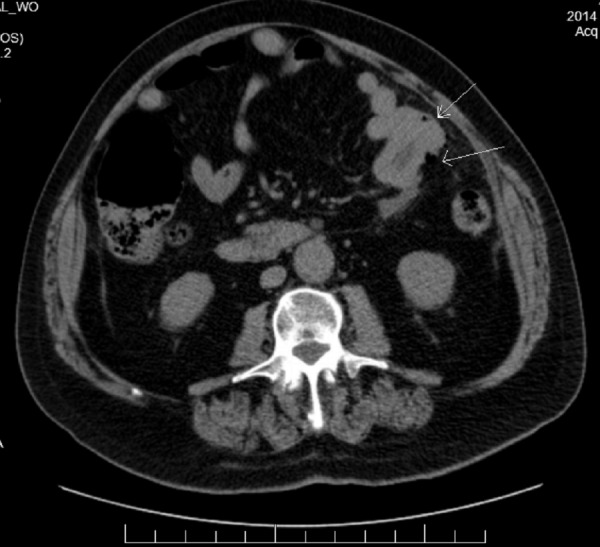
CT of abdomen showing evidence of thickened bowel as pointed with white arrows, along with pockets of free peritoneal air.

He was initiated on ipilimumab and radiotherapy to the brain and discharged after clinical stabilization. He presented to the hospital three months later with an acute onset of severe abdominal pain accompanied by significant watery diarrhea for three days. By this time, he had completed three cycles of ipilimumab therapy and was put on prednisone in the office. A CT scan of his abdomen revealed free peritoneal air, indicative of perforated viscus (Figure 4).

An urgent exploratory laparotomy was performed, and a mass was found on the antimesenteric border of the duodenum with small areas of perforation with surrounding fibrinous exudation. The perforated area was resected, and a functional end-to-end anastomosis was created. Unfortunately, the patient had a postoperative intracranial bleed and was referred to palliative care.

## Discussion

Our case description portrays the classic features of how a malignant melanoma may insidiously metastasize widely and on occasion present as a catastrophic complication. It is one of the more common primary malignancies that spreads to the GI tract [3]. Malignant melanoma involvement in the GI tract varies markedly with regards to its presentation. Various studies have quoted the incidence of symptomatic GI metastatic melanoma to be from 0.8% to 4.7% [4-5] while the proportion of GI involvement in postmortem analyses of disseminated melanoma is substantially greater (60%) [1]. Our case offers a rare illustration of both the occult and manifest gastrointestinal involvement of melanoma.

The majority of melanoma involvement in the GI tract is considered to be metastatic, and the definition of primary GI melanoma is controversial [6]. The most common primary location of melanoma with GI metastasis is the extremities (15% to 57%) followed by the trunk (13% to 54%) and head and neck (5% to 33%). The origin remains unknown in 10% to 26% of cases [7]. Moreover, the propensity of the primary tumor to metastasize is dependent on Clark staging, with >70% of Clark level III (as in our case) and Clark level IV lesions involving the GI tract.

The small bowel is the most common location for metastasis (35% to 97%) followed by the stomach, duodenum, (5% to 50%) and colon (5% to 32%) [8]. The average reported time from initial diagnosis to intestinal metastasis ranges from 21.6 to 54 months [8]. This case fell within this range at 35 months. Vague chronic abdominal pain is the most common presentation followed by GI bleeding and weight loss, making it difficult to diagnose. Acute abdomen due to perforation, intussusception, or obstruction is a rare clinical presentation [9]. 

The appearance of metastasis on endoscopy may take the form of ulcers, nodules, or polyps, which may be pigmented or amelanotic. Diagnostic yield of biopsy from the margins of ulcerations is >90% [1]. Involvement of the serosa and mesentery is not uncommon. Therefore, the endoscopic assessment may be falsely negative.

Of note, the anti-CTLA-4 antibody ipilimumab used in the treatment of malignant melanoma has been associated with severe GI adverse effects, including perforation. The incidence of small bowel perforation in patients on ipilimumab may be as high as two percent [10]. The fact that our patient was undergoing treatment with anti-CTLA-4 antibody may have been a predisposing factor in the etiology of perforation.

The literature suggests that if the GI tract is the only location of metastases, surgical resection can lead to substantial survival benefit, which is marked if resection is complete on microscopic examination [4,8]. This makes following up on the disease very important. We found no evidence of survival benefit in asymptomatic patients who underwent resection. In contrast, there was an overwhelming improvement in survival (48.9 months vs. 5.4 months) in symptomatic patients who underwent resection [4]. Unfortunately, our patient had evidence of disease in the brain, lung, bowel, and liver, making him a poor candidate for resection after the jejunal disease was found on initial presentation.

Further treatment options other than surgical resection include immunotherapy, chemotherapy or biochemotherapy [3]. Prognosis for malignant melanoma is largely dependent on the extent of the disease at the time of diagnosis. However, melanoma involvement in the GI tract is a marker of poor prognosis overall, with a five-year survival rate of 14% and a median survival of 12.5 months.

## Conclusions

Malignant melanoma has been called the “syphilis of modern oncology” due to its myriad presentations. Metastasis to the bowel may be devoid of clinical symptoms, which may be the reason that it is found as a bystanding lesion in autopsies of melanoma patients. Rarely, however, malignant melanoma metastasizing to the bowel may lead to perforation and present as an acute abdomen. The index of suspicion should be high in patients who have a prior history of malignant melanoma. Furthermore, immunotherapy with ipilimumab may be associated with an increase in the incidence of bowel perforation in patients who already have evidence of GI metastasis. Further studies to elucidate the numerical risk of such an event are needed. Reporting such a rare entity may help in providing further data that will help improve the management of such cases.
